# A Novel Cellulose-Based Polymer for Efficient Removal of Methylene Blue

**DOI:** 10.3390/membranes10010013

**Published:** 2020-01-10

**Authors:** Diana Gago, Ricardo Chagas, Luísa M. Ferreira, Svetlozar Velizarov, Isabel Coelhoso

**Affiliations:** 1 LAQV-REQUIMTE, NOVA School of Science and Technology, 2829-516 Caparica, Portugal; dx.gago@campus.fct.unl.pt (D.G.); lpf@fct.unl.pt (L.M.F.); s.velizarov@fct.unl.pt (S.V.); 2 i3N/CENIMAT, Department of Materials Science, NOVA School of Science and Technology, 2829-516 Caparica, Portugal; r.chagas@fct.unl.pt

**Keywords:** adsorption isotherms, adsorption kinetics, dicarboxymethyl cellulose, dye removal, membrane filtration

## Abstract

A novel cellulose-based cross-linked polymer, dicarboxymethyl cellulose (DCMC), has been synthesized and used for methylene blue (MB) removal. Inductively coupled plasma atomic emission spectrometry (ICP-AES), Fourier-transform infrared spectroscopy (FTIR), nitrogen porosimetry, and optical microscopy were employed to characterize the structure of the cellulose-based adsorbent. The number of carboxylate groups per gram of polymer (CG) was calculated with sodium content determined by ICP-AES. Systematic equilibrium and kinetic adsorption studies were performed to assess the polymer suitability for dye removal. The effect of pH on its adsorption capacity was also studied and the equilibrium adsorption data was analyzed using Langmuir, Freundlich, and Sips isotherms. At pH = 3, the adsorption isotherms followed the Langmuir model with a maximum adsorption capacity of 887.6 mg/g. At pH = 6.4, the adsorption isotherms produced S-shape curves and were best fitted with the Sips model. The maximum MB uptake increased to 1354.6 mg/g. Pseudo first-order and second-order models were used to fit the kinetic data. A pseudo second-order kinetic model provided the best correlation for the adsorption of MB onto DCMC. Adsorption coupled with membrane filtration achieved 95% methylene blue removal and DCMC can be successfully regenerated and reused in consecutive experiments.

## 1. Introduction

Dyes and pigments are used in several chemical industries, such as pharmaceutical and textile. These industrial processes require large volumes of water and are responsible for the annual release of up to 150,000 tons of dyes into wastewaters [1]. The presence of dyes in wastewaters is easily recognized by human eye, due to a change of color, and may have dire effects on aquatic life and compromising photosynthetic activity of certain aquatic species [2]. Moreover, these pollutants are generally resistant to biodegradation because of their complex aromatic structures [2]. Methylene blue is a common representative of cationic dyes [3]. It is typically used in the textile industry, for dying cotton and silk [4]. Numerous reports are available in the literature concerning the removal of this specific dye. Environmental awareness and regulatory measures contribute to a growing interest on cheaper and more effective techniques for the treatment of wastewaters containing dyes [1,3,4].

Many techniques have been tested for dye removal and adsorption is considered the most efficient process due to its low cost, simplicity, and lack of formation of harmful byproducts [1,4,5]. Ion exchange is also commonly used in wastewater treatment [6,7]. Activated carbons are used as adsorbents due to their efficiency in dye removal. However, their high production cost urges the research on alternative economical and renewable adsorbents [4,8,9].

Cellulose is the most abundant organic material in nature. It is biodegradable and renewable. Cellulose-based materials are commonly used in various applications, namely as adsorbents in wastewater treatment [10,11,12]. The development of new cellulose-based polymers is motivated by the demand of low-cost and environmentally friendly materials [10].

Dicarboxymethyl cellulose (DCMC) is prepared by reaction of sodium 2-bromomalonate with cellulose [13,14]. Malonic acid has pKa values of approximately 2.85 and 5.70, which the cellulose bond should not greatly influence, resulting in mainly deprotonated acid groups for pH greater than 3 [15]. For this reason, DCMC can perform ion exchange at low pH [16]. The polymer works as an anionic polyelectrolyte, suitable for the removal of positively charged synthetic dyes due to its active functional groups, such as hydroxyl and carboxyl [16,17].

In this work, the potential of cross-linked dicarboxymethyl cellulose for removal of methylene blue as a cationic dye model was investigated. The synthesized polymers were characterized using inductively coupled plasma atomic emission spectrometry (ICP-AES) and Fourier-transform infrared spectroscopy (FTIR). Influence of parameters, such as solution pH and number of carboxylate groups per gram of polymer, were studied. Adsorption equilibrium and kinetic studies were performed. Equilibrium adsorption isotherms are essential in understanding the interactions between adsorbent and adsorbate, providing information on surface properties and adsorption capacities [5,8,18,19,20]. Langmuir isotherm assumes that adsorption occurs without lateral interactions between adsorbed molecules, producing homogeneous binding sites and a monolayer coverage of the outer surface of the adsorbent [8,21,22]. Freundlich isotherm states that adsorption occurs on a heterogeneous surface, where binding strength decreases with increasing occupation [8,21]. Sips isotherm is a combination of the Langmuir and Freundlich isotherm models [23,24]. Kinetic studies give better understanding of the adsorption process, namely adsorbent behavior in the presence of distinct adsorbates [3,19,25]. Lagergren first described adsorption kinetics of liquid-solid systems based on solid capacity [26,27]. Lagergren’s pseudo first-order (PFO) equation is based on the assumption of physisorption process [28]. The PFO model assumes that the sorption rate decreases linearly as the sorption capacity increases [12]. The pseudo second-order (PSO) model is similar to PFO, but it suggests a chemisorption process [28,29].

Membrane filtration is increasingly used in water treatment processes [30,31]. However, disadvantages such as membrane fouling often contribute to reduced process performance over time. To minimize such limitations, possible membrane-solute interactions should be avoided [30]. Therefore, the use of adsorption coupled with membrane filtration has great potential in wastewater treatment, since it can prevent direct solute(s) contact with the membrane, thus minimizing its fouling [31,32]. In this work, adsorption was coupled with filtration in a MET^®^Cell Dead-End system using ultrafiltration membranes, such as Microdyn Nadir UV150 and UC500. Adsorbent and membrane reusability studies were also performed.

## 2. Materials and Methods

### 2.1. Materials

Air-dry cellulose (MN 400 Avicel) was obtained from Macherey-Nagel. Sodium bromomalonate was previously synthesized by LAQV-REQUIMTE with bromine and malonic acid. A standard citrate buffer solution (25 mM, pH = 3) was prepared with sodium citrate dihydrate (*MW* = 294 g/mol) and citric acid monohydrate (*MW* = 210 g/mol) to be used as a buffer. Deionized water without any buffer was used to prepare methylene blue solutions with a pH = 6.4. Separate stock solutions with a concentration of 2 and 3 g/L were prepared by dissolving the required amount of methylene blue in deionized water or citrate buffer. The solutions were diluted with deionized water or citrate buffer to achieve concentration and pH values needed for the experiment. Other chemicals and solvents were laboratory grade and used without further purification.

### 2.2. Synthesis of Dicarboxymethyl Cellulose

**Figure 1:**
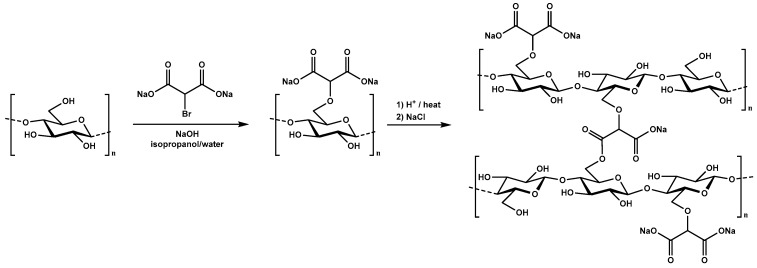
Schematic representation of the polymer synthesis including the heterogeneous grafting of the malonic moiety in the cellulose backbone [14] and crosslinking by intra/intermolecular esterification [13].

Cross-linked dicarboxymethyl cellulose (DCMC) was synthesized specifically for this work. Three different polymers were prepared differing in the amount of sodium 2-bromomalonate (BMA) added. To produce polymers with varying number of carboxylate groups, 1, 2, or 3 molar equivalents of BMA were added per anhydroglucose units (AGU), resulting in DCMC 1, DCMC 2, or DCMC 3 respectively. DCMC was synthesized following the procedure described by Ferreira et al. [13,14]. Figure 1 presents the schematic representation of the synthesis of dicarboxymethyl cellulose.

A total of 5 g of air-dry cellulose and 175 mL isopropanol were stirred vigorously and 5.5 mL of water with 3.7 g of NaOH was slowly added to the mixture for 10 min at room temperature. The mixture was magnetically stirred for 1 h and the appropriate quantity of sodium 2-bromomalonate in 18 mL of water was added. After complete dispersion, the mixture was placed on a water bath at 60 °C for 3 h with vigorous stirring. After this time, the reaction mixture was filtrated, and the solid suspended in 70% (*v*/*v*) methanol and neutralized with acetic acid. Aqueous and pure methanol were used for further purification of the product. Finally, the product dried under vacuum at room temperature. The latter was protonated dispersing the powdered product in 20% sulfuric acid solution for 1 h. The product was decanted, and the precipitate washed with distilled water until neutral pH. After drying, the protonated polymer was heated at 100 °C for 1 h promoting its cross-linking by esterification (formation of ester bonds between the carboxylic acid of the malonate group and the hydroxyl group of adjacent cellulose chains). The resulting cross-linked polymer was washed with 1 M NaCl until neutral pH followed by washing with distilled water to remove remaining NaCl. The sodium salt of the cross-linked polymer was isolated by filtration and dried under vacuum yielding a white powder. Table 1 shows the appropriate quantities of each reagent for the synthesis of dicarboxymethyl cellulose in these conditions.

### 2.3. Characterization of Dicarboxymethyl Cellulose

Characterization methods were performed to determine the influence of stoichiometry on chemical structure. Differences in sodium content were assessed by ICP-AES. For it, sodium dicarboxymethyl cellulose was first purified by dialysis against deionized water. Dry dialyzed samples were hydrolyzed for ICP analysis by adding 500 µL nitric acid to a known mass of polymer (approximately 1.0 mg). Then, they were incubated at 70 °C for 1 h and analyzed on a Horiba Jobin-Yvon Ultima model equipped with a 40.68 MHz RF generator, a Czerny–Turner monochromator with 1.00 m (sequential), and an autosampler AS500 (Horiba, Kyoto, Japan). The resulting percentage of sodium is equivalent to the number of carboxylate functional groups able to perform the desired cation exchange. Percentage of sodium in each sample is defined as the sodium to polymer mass ratio (*w*/*w*). The number of carboxylate groups per gram of polymer (CG) was calculated by Equation (1).

(1) $$CG=\frac{\frac{\%Na}{100}\times 1000}{23}$$

where the term “23” represents the molecular mass of sodium.

FTIR spectra of the samples were recorded on a Perkin-Elmer FT-IR Spectrometer Spectrum Two (Waltham, MA, USA), equipped with an attenuated total reflection (ATR) cell, in the range of 4000–400 cm^−1^. The pore size distribution was determined by nitrogen adsorption-desorption experiments at 196 °C (77 K), using a Micromeritics ASAP 2010 instrument (Micromeritics, Norcross, GA, USA). The Brunauer–Emmett–Teller (BET) method was used to calculate specific surface area. Morphology and size distribution of dicarboxymethyl cellulose were analyzed by optical microscopy with a Nikon Eclipse ci (Tokyo, Japan). The images were processed with ImageJ software (National Institutes of Health, Bethesda, MD, USA).

### 2.4. Adsorption Experiments of Methylene Blue

The influence of number of carboxylate groups per gram of polymer was studied at pH = 3. The process was also studied at pH = 6.4 (DI water) for the polymer with higher adsorption capacity at pH = 3. Two mL of methylene blue solutions with concentrations between 40 and 3000 mg/L were added to 4 mg of polymer and kept in a water bath for 48 h and at room temperature (25 °C). A Spectronic Helios Alpha spectrometer (Thermo Electron, Waltham, MA, USA) was used to determine methylene blue concentrations at 664 nm, by comparison with a calibration curve in the range of 0–4 mg/L prepared with methylene blue and deionized water or citrate buffer. The experiment was performed in triplicate and mean values are presented. The adsorption capacity is calculated by Equation (2).

(2) $$q=\frac{\left(C_{0}-C_{e}\right)}{m}V$$

where *q* (mg/g) is adsorption capacity; *C*_0_ and *C_e_* (mg/L) are the initial and equilibrium concentrations of methylene blue in the solution, respectively; *V* (L) is solution volume and *m* (g) is adsorbent mass.

### 2.5. Modelling of Adsorption Isotherms

Adsorption isotherms describe interactions between adsorbate and adsorbent [22]. In the present work, Langmuir, Freundlich, and Sips models were applied and compared for rationalization of the obtained experimental data for equilibrium adsorption of methylene blue onto dicarboxymethyl cellulose.

The Langmuir isotherm is expressed by Equation (3) [8,21].

(3) $$q=\frac{q_{m}C_{e}}{K_{d}+C_{e}}$$

where *q_m_* (mg/g) is the maximum adsorption capacity, *C_e_* (mg/L) is equilibrium concentration, and *K_d_* (L/g) is the Langmuir adsorption equilibrium constant, representing the affinity between adsorbate and binding sites.

The Freundlich mathematical model is expressed by Equation (4) [8,21].

(4) $$q=KC_{e}^{1/n}$$

where *K* (L/mg) is the Freundlich constant, which relates to adsorption capacity, *C_e_* (mg/L) is equilibrium concentration and *n* is the heterogeneity factor. Larger values of *n* indicate stronger adsorbate-adsorbent interaction and it is generally stated that values of *n* in the range of 1–10 are indicative for favorable adsorption [26].

The Sips equation is given by Equation (5) [23,24,29,33,34].

(5) $$q=\frac{q_{m}K_{s}C_{e}^{1/n_{s}}}{1+K_{s}C_{e}^{1/n_{s}}}$$

where *q_m_* (mg/g) is maximum adsorption capacity, *K_s_* (mg/L)^−1/n^_s_ is the Sips equilibrium constant, *C_e_* (mg/L) is equilibrium concentration, and 1/*n_s_* is the heterogeneity factor.

### 2.6. Kinetic Adsorption Experiments and Modelling

Experiments were performed with 2 mL methylene blue solutions with concentration of 4 mg/L and 4 mg of DCMC, at pH = 3.0 and 6.4, respectively. Pseudo first-order and pseudo second-order nonlinear kinetic models were used to fit the experimental data.

Pseudo first-order model is expressed by Equation (6) [25,35].

(6) $$q=q_{m}\left(1-e^{-K_{1}t}\right)$$

where *q_m_* (mg/g) is maximum adsorption capacity, *K_1_* (min^−1^) is the rate constant, and *t* (min) is time.

The Pseudo second-order model follows Equation (7) [25,35].

(7) $$q=\frac{q_{m}^{2}K_{2}t}{1+q_{m}K_{2}t}$$

where *q_m_* (mg/g) is maximum adsorption capacity, *K_2_* (mg g^−1^ min^−1^) is the rate constant, and *t* (min) is time.

### 2.7. Adsorption Coupled with Filtration

**Figure 2:**
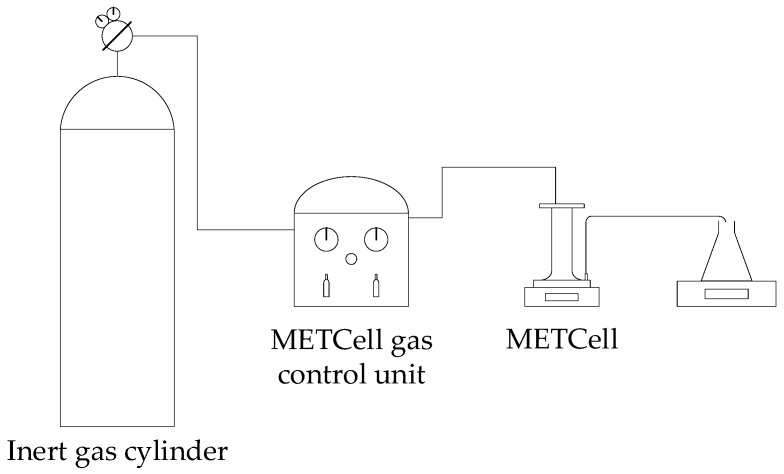
Schematic diagram of the MET^®^Cell filtration system.

Adsorption coupled with filtration experiments were performed using a MET^®^Cell Dead-End filtration system (Evonik Membrane Extraction Technology Ltd, London, UK) with a porous membrane disk with 8.1 cm of diameter and active filtration area of 51.5 cm^2^ under constant pressure mode. The transmembrane pressure was monitored by a pressure transducer connected at the cell inlet. The permeate flux was determined by measuring the permeate weight with an electronic balance, connected to a computer for continuous data acquisition. All tests in this study were performed with no fluid agitation. Figure 2 presents a schematic diagram of the experimental setup for membrane filtration.

Preliminary filtration experiments using commercial membranes of PVDF and regenerated cellulose (Microdyn Nadir UV150 and UC500, respectively) were performed. A total of 200 mL of a 4 mg/L methylene blue aqueous solution were added to the system. The filtration process was conducted at room temperature without agitation. An ultrafiltration membrane (UV150 or UC500) with a molecular weight of 150 or 500 kDa, respectively, was placed at the bottom of the MET^®^Cell and supported by a porous stainless-steel disk. The system was operated under argon pressure of 5 bar in order to permeate the liquid through the membrane. Permeate samples were collected and analyzed spectrophotometrically to determine methylene blue concentrations.

**Figure 3:**
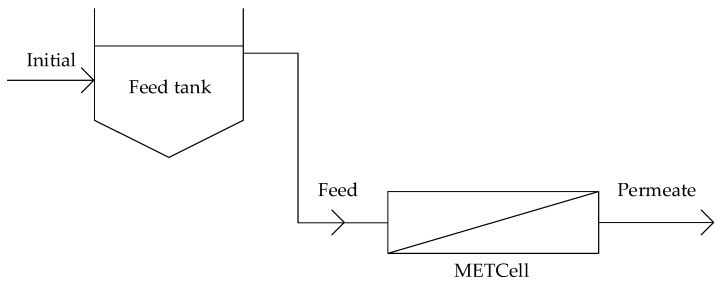
Schematic diagram of adsorption coupled with filtration treatment.

Adsorption coupled with filtration experiments were performed by adding 400 mg of DCMC 3 to 200 mL of a 4 mg/L methylene blue aqueous solution. The adsorption process was conducted at room temperature. After 30 min, the mixture was decanted and added to the MET^®^Cell system. The ultrafiltration membranes (UV150 or UC500) were used in these experiments as described previously. Figure 3 presents a schematic diagram for the adsorption coupled with filtration treatment.

Reusability studies were performed on the polymer and on the used membranes using deionized water and 1 M NaCl aqueous solution as eluent agent. After adsorption, the polymer was washed with deionized water and decanted. The membranes were then air dried at room temperature. Both materials were immersed in deionized water and in the NaCl solution for 3 h for desorption of dye molecules and the solutions were analyzed spectrophotometrically to determine methylene blue concentrations.

## 3. Results and Discussion

### 3.1. Characterization of Dicarboxymethyl Cellulose

The content of sodium in dicarboxymethyl cellulose samples was determined by ICP-AES. The results obtained are presented in Table 2. As expected, with increasing sodium 2-bromomalonate content used in the polymer synthesis there is an increase in this number.

**Figure 4:**
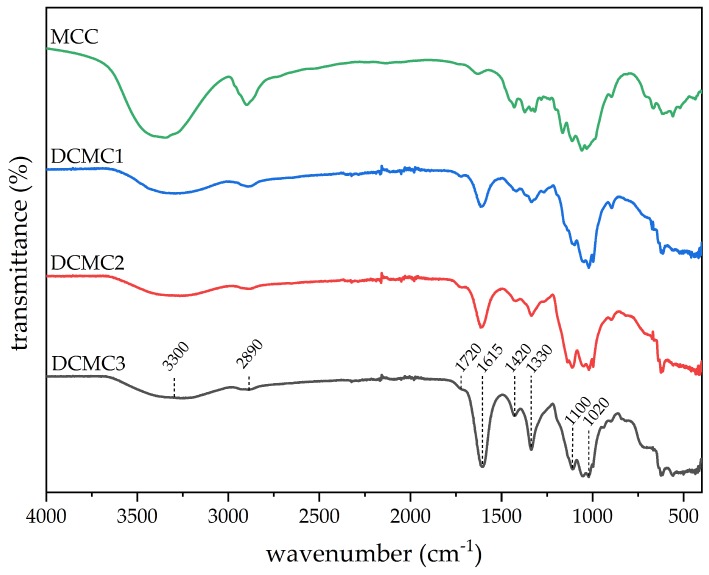
Attenuated total reflection Fourier-transform infrared spectroscopy (FTIR-ATR) spectra of microcrystalline cellulose (MCC) and dicarboxymethyl cellulose polymers (DCMC 1, DCMC 2, and DCMC 3).

FTIR was used to characterize the chemical structure and functional groups of the products. Figure 4 shows the absorption spectra of microcrystalline cellulose and of the three different dicarboxymethyl cellulose polymers prepared.

A broad adsorption peak at 3300 cm^−1^ is in the range of –OH stretching vibration [22,36]. A decrease of intensity with a higher number of carboxylate groups (CG) could be justified by the replacement of several hydroxyl groups by the new carboxylate groups. The peak at 2890 cm^−1^ is attributed to the C–H stretching vibration [22,36]. The intensity of a band at 1720 cm^−1^, which is related to the carbonyl stretching of the ester groups, increased with number of carboxylate groups per gram of polymer since the ester groups are formed during the cross-linking procedure. With a higher number of carboxylic acid groups an increase in the number of ester groups can be expected. The bands at 1615, 1420, and 1330 cm^−1^ are attributed to COO– asymmetric stretching, COO– symmetric stretching, and C–O stretching, respectively [36]. The asymmetric band increases in the presence of carboxylate groups (COO–), which explains the increase in intensity with a higher number of carboxylate groups per gram of polymer. Strong broad peaks at approximately 1100 and 1020 cm^−1^ indicate the presence of C–O–C bonds, characteristic of the cellulose backbone [12].

Porosimetry of dicarboxymethyl cellulose was investigated by N_2_ adsorption-desorption isotherms indicating a nonporous material. Specific surface areas calculated by the Brunauer–Emmett–Teller (BET) method varied between 3.71 and 5.05 m^2^/g.

**Figure 5:**
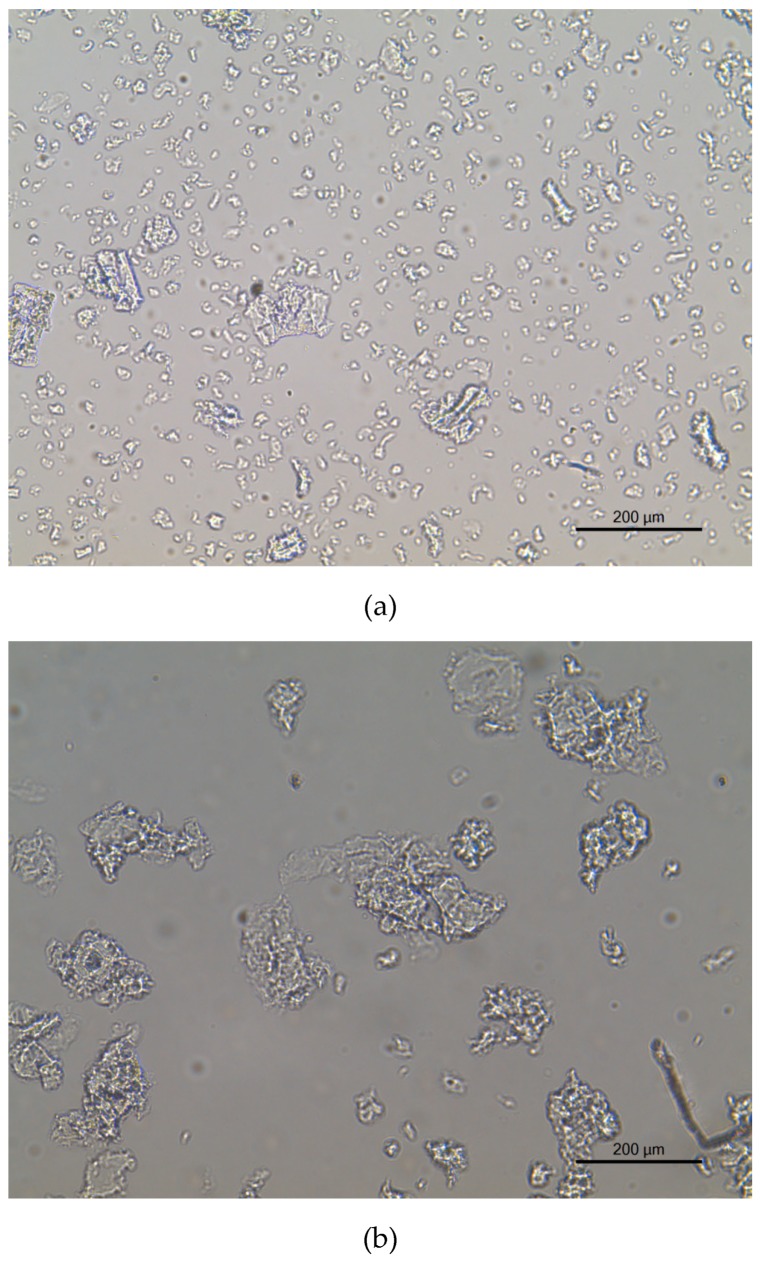

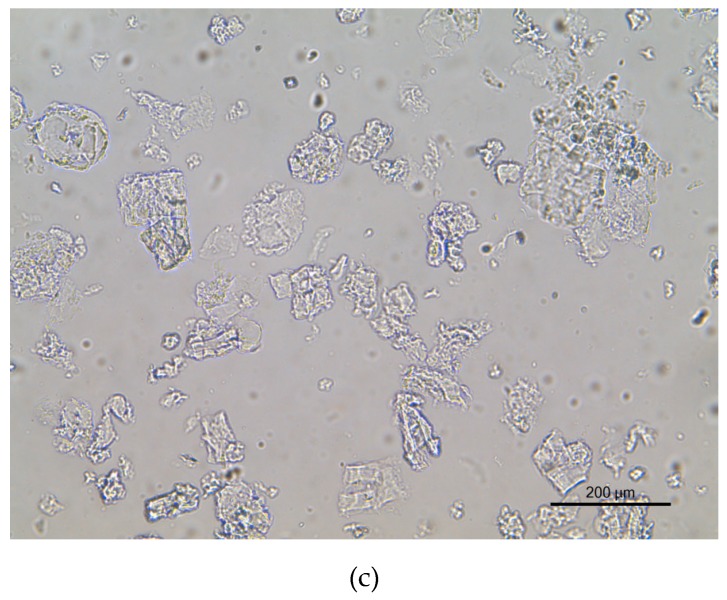
Optical microscopy images of dicarboxymethyl cellulose in aqueous solution: (**a**) DCMC 1, (**b**) DCMC 2, and (**c**) DCMC 3.

The images obtained by optical microscopy are shown in Figure 5. To disrupt aggregation, the polymer was mixed with deionized water. Based on this figure, the polymer does not have a defined shape. Analyzing the images with ImageJ, the particle size and surface area varies with the number of carboxylate units per gram of DCMC. The calculated dimensions for the polymers are presented in Table 3. Using ImageJ, length was measured by extending a line for the length of the polymer. Surface area was calculated by the software when delimiting the particles. DCMC 1 is considerably smaller than DCMC 2 and DCMC 3. Contrarily, there are no significant differences in the dimensions of DCMC 2 and DCMC 3.

### 3.2. Adsorption Isotherms

#### 3.2.1. Effect of Number of Carboxylate Groups

Adsorption experiments were carried out with the polymer with different numbers of carboxylate groups (CG) per gram of DCMC at pH = 3.

**Figure 6:**
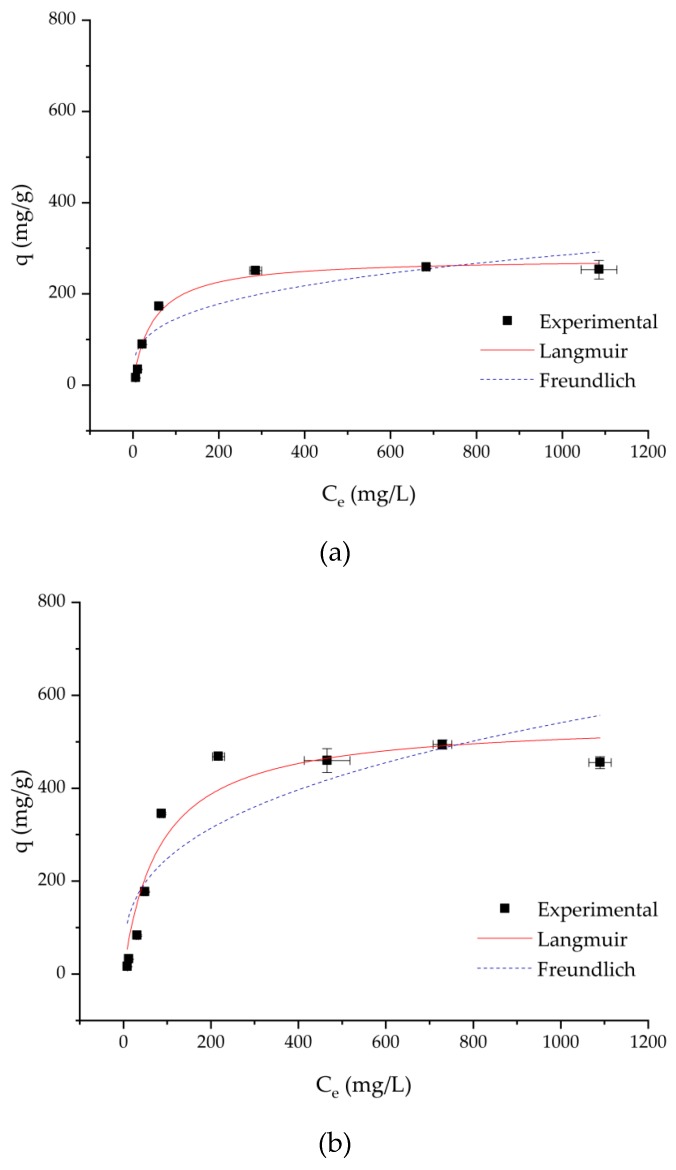

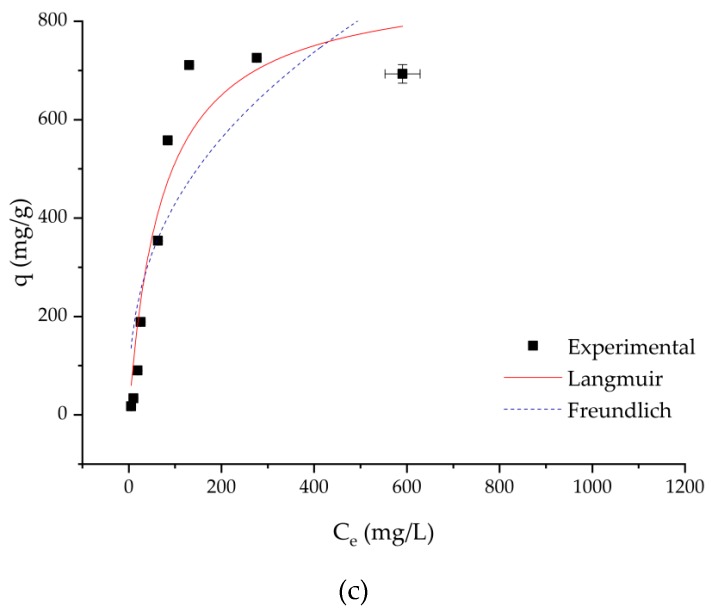
Adsorption isotherms at pH = 3: (**a**) DCMC 1, (**b**) DCMC 2, and (**c**) DCMC 3.

Figure 6 illustrates the effect of the number of carboxylate groups per gram of DCMC on the adsorption isotherms at pH = 3. The adsorption capacity increases with increasing methylene blue concentration. Saturation capacity for all DCMC samples was close to 200 mg/L of methylene blue. With increasing number of carboxylate groups, the rise to saturation is steeper, which is justified by a higher availability of binding sites.

Table 4 presents adsorption isotherm parameters calculated with fitting of the data to the Langmuir and Freundlich models. Based on the obtained correlation coefficient values (*R*^2^) presented in Table 4, it becomes clear that the Langmuir model provides a better description of the experimental data. The *R*^2^ values obtained from the Freundlich model are 0.841, 0.789, and 0.767 for DCMC 1, DCMC 2, and DCMC 3 at pH = 3, respectively. Using the Langmuir isotherm model, the correlation values were much higher (*R*^2^ > 0.9). Thus, the applicability of the Langmuir model is consistent with the plateaus observed in Figure 6, indicating a monolayer adsorption process without lateral interactions between adsorbed molecules [19]. This is in agreement with the literature, showing the applicability of the Langmuir model to ion exchange isotherms [7]. Maximum adsorption capacities calculated from the Langmuir model reached 277.6, 546.2, and 887.6 mg/g for DCMC 1, DCMC 2, and DCMC 3 at pH = 3, respectively (see Table 4). These values were all close to the experimental values, thus reinforcing the statement that the Langmuir model is the most applicable fitting model to the investigated case.

#### 3.2.2. Effect of pH

The pH of the dye solution is an important factor for the adsorption process, influencing the surface charge of the adsorbent, which consequently affects adsorption capacity [37,38]. DCMC 3 provided better results for the adsorption experiment at pH = 3, compared with the other samples. In order to investigate the effect of pH on methylene blue uptake, experiments were performed using deionized water at pH = 6.4 with DCMC 3. In this case, no buffer was used to simulate a real situation of a wastewater containing just methylene blue.

**Figure 7:**
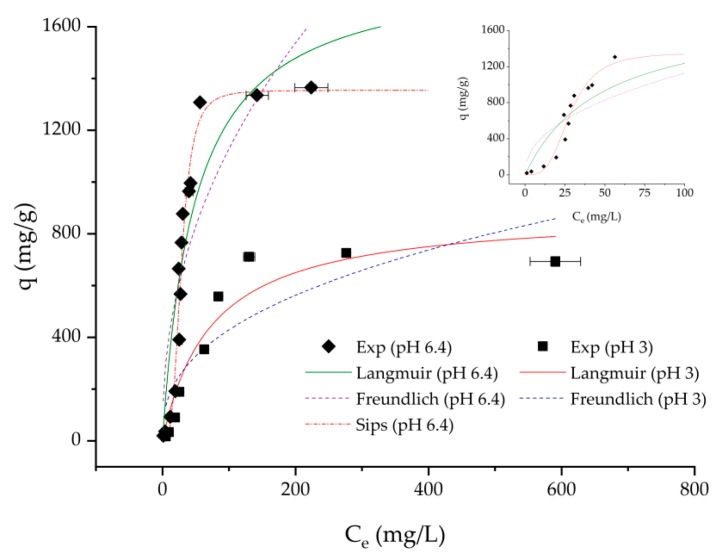
Effect of pH on adsorption isotherm of methylene blue in DCMC 3.

Figure 7 is characterized by S-shaped adsorption. These types of isotherms are usually associated with cooperation adsorption caused by solute–solute attraction and/or competing reaction with the solution, which inhibit solute adsorption [39].

Figure 7 shows that the maximum removal of methylene blue is achieved at pH = 6.4. With increasing pH, negatively charged binding sites increase thus favoring adsorption of cationic dyes, such as methylene blue. Conversely, at pH = 3.0 dicarboxymethyl cellulose has an increase in H^+^ ion concentration resulting in electrostatic repulsion between the polymer and the cationic dye. Due to the presence of a low pKa ether group, dicarboxymethyl cellulose can perform ion exchange at low pH (2.5–3.5) [13]. With an increase of solution pH above the pKa, the binding sites are increasingly deprotonated. A solution pH above the pKa of the polymer’s functional groups promotes maximum methylene blue removal by reducing the competition between adsorbing dye molecules. In the experiments with solution pH = 3.0, dicarboxymethyl cellulose is only partly deprotonated therefore reducing available binding sites for the adsorption of methylene blue.

Since experimental data at pH = 6.4 did not adjust well to Langmuir and Freundlich models, the results were fitted with the Sips model, a combination between the previous models [40]. Based on the correlation factor (*R*^2^ = 0.968), the Sips model described the experimental data more appropriately. The calculated Sips heterogeneity factor *n* is 0.3 and equilibrium constant *Ks* is 8.794 × 10^−6^ (mg/L)^1/n^_s_. This calculated maximum adsorption capacity is very similar to the experimentally obtained values of 1354.6 and 1365.5 mg/g, respectively.

Figure 7 illustrates the effect of pH on adsorption isotherms of methylene blue onto the developed cellulose-based adsorbent. Adsorption rate and adsorption capacity at pH = 6.4 were significantly higher than at pH = 3.0. Maximum adsorption capacities for the best fitting models went from to 887.6 to 1354.6 mg/g for pH = 3.0 and 6.4, respectively. As mentioned previously, higher methylene blue uptake for higher pH is expected due to the increasing availability of binding sites.

#### 3.2.3. Comparison with Other Adsorbents

Comparison of methylene blue adsorption on other adsorbents reported in literature is presented in Table 5. Adsorption capacity of dicarboxymethyl cellulose was found higher than those reported in literature, thus confirming that this novel cellulose-based adsorbent is promising for removal of methylene blue from aqueous solutions.

Commercial activated carbon (CAC) is commonly used in wastewater treatment [41]. CAC-based materials are efficient adsorbents but have high production and regeneration costs [8,9]. Alternatively, DCMC is synthesized from an abundant biodegradable source, resulting in an economical adsorbent with facile disposal. The results obtained in the present study showed that DCMC possesses adsorption capacities in the same order of magnitude as CACs. Therefore, dicarboxymethyl cellulose may be successfully used as a water remediation tool, including the removal of methylene blue from aqueous solutions.

### 3.3. Adsorption Kinetics

**Figure 8:**
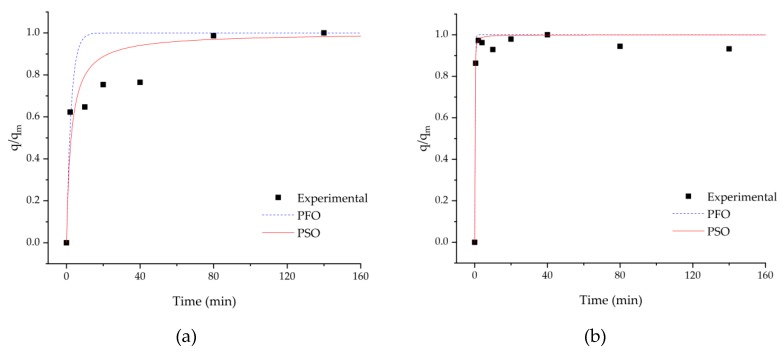
Adsorption kinetics of a 4 mg/L solution represented by normalized adsorption capacity: (**a**) pH = 3 and (**b**) 6.4.

Kinetic studies were performed at pH = 3.0 and 6.4, with initial methylene blue concentration of 4 mg/L. Changes in solution concentration over time can be observed in Figure 8.

Figure 8 shows the effect of pH on methylene blue adsorption kinetics at a 4 mg/L initial concentration. For the same concentration, an increase in pH results in a higher dye uptake, consistent with previous results. As shown in this figure, the adsorption of methylene blue onto DCMC occurred within the first 30 s and there is almost complete dye removal after 1 h. After this rapid adsorption, an equilibrium is established.

Experimental data on the adsorption of methylene blue onto dicarboxymethyl cellulose was fitted by pseudo first-order and pseudo second-order kinetic models. The estimated adsorption kinetic parameters are summarized in Table 6.

The data shows high correlation factors (*R*^2^) for both kinetic models. However, at pH = 3 the experimental data is better fitted by a pseudo second-order model. The calculated adsorption capacity values from PSO are close to the experimental results, suggesting the applicability of this model for methylene blue adsorption kinetics on dicarboxymethyl cellulose.

From Table 6, it can be observed that the adsorption capacity increases with increasing pH. Adsorption capacity of methylene blue onto the polymer changes from 0.881 to 3.134 mg/g, when pH is increased from 3.0 to 6.4. Additionally, the kinetic rate constant K_2_ decreases with increasing pH. At a lower pH, there are less negatively charged binding sites, thus increasing the competition between dye molecules for adsorption on the polymer’s surface, thus lowering kinetic rates.

### 3.4. Adsorption Coupled with Filtration

Filtration experiments were carried out on the MET^®^Cell system using an ultrafiltration membrane (Microdyn Nadir UV150 or UC500). The system was filled with 200 mL of a 4 mg/L methylene blue solution. The solution was permeated by using a transmembrane pressure of 5 bar. Table 7 shows the methylene blue removal efficiency achieved in these experiments. Both membranes allowed for high removal efficiencies with over 85% methylene blue removal. Microdyn Nadir UV150 and UC500 have a pore size of 150 and 500 kDa, respectively, nearly three orders of magnitude higher than the molar mass of methylene blue (319.85 Da). For this reason, complete removal of the cationic dye was not expected. Filtration of 200 mL of MB solution with UC500 was slower than with UV150 (140 and 20 s, respectively), even though the former has a larger pore size. A slower process with the UC500 membrane may be attributed to its composition, since regenerated cellulose may be adsorbing the cationic dye [34]. The accumulation of methylene blue on the membrane may also justify the slightly higher removal efficiency.

Since process requirements and regulations may demand higher purity of the permeate solutions, an adsorption process coupled with membrane filtration was tested. In this experiment, 200 mL of a 4 mg/L methylene blue solution were added to 400 mg of polymer. After 30 min, the solution was decanted and added to the MET^®^Cell system where an ultrafiltration membrane (Microdyn Nadir UV150 or UC500) had previously been placed. A transmembrane pressure of 5 bar was induced to permeate the solution through the membrane. Table 8 presents the results obtained regarding the methylene blue removal after adsorption and subsequent membrane filtration. In both experiments, close to 60% methylene blue removal was achieved within the first 30 min. After the decantated solution was filtered, the permeate was visually clear. The spectrophotometric measurements showed only residual (less than 10 ppb) methylene blue concentrations for the experiment using either membrane. Methylene blue removal after the adsorption and filtration process was close to 95%. However, the filtration process lasted approximately half an hour. Therefore, this treatment does not achieve significantly better removal efficiencies, when compared to the filtration process, while requiring a total of 1 h instead of 2 min. For this reason, adsorption coupled with filtration does not appear a feasible option for methylene blue removal, since it is more time-consuming and less cost-effective.

Finally, reusability experiments were performed both for the polymer and the membranes used. The polymer was washed with deionized water after adsorption and decanted while the membranes were air dried at 30 °C to avoid diluting the desorbing solution. Then, both materials were immersed in deionized water and in 1 M NaCl for 3 h. Elution with water has not occurred in both polymer and membranes. DCMC presented desorption efficiency with NaCl solution of 93.9%, whereas none of the membranes desorbed the sequestered dye. This high desorption efficiency suggests that the polymer may be reused in consecutive cycles, thus contributing to a more sustainable and economical process. On the other hand, since the membranes did not desorb methylene blue, their repeated use is constrained. Incorporation of DCMC in porous membranes would allow to couple adsorption and filtration providing reuse of the membranes, as well.

## 4. Conclusions

In this study, cross-linked dicarboxymethyl cellulose prepared from air-dried cellulose was used for the adsorption of methylene blue from aqueous solutions. Adjusting the number of sodium 2-bromomalonate (BMA) equivalents in the synthesis increased functionalization. FTIR and ICP provided information on the effect of BMA equivalents in the chemical modification of the polymer. As expected, increasing BMA molar equivalents increases the number of carboxylate groups per gram of polymer (CG). Experimental results showed that methylene blue adsorption was dependent on adsorbent surface characteristics, which are dependent on the solution pH. At an acidic pH, the Langmuir isotherm model adjusted the experimental data better, suggesting monolayer adsorption on a homogenous adsorbent surface. The maximum dye uptake at these conditions was 887.6 mg/g. At pH = 6.4, experimental data fitted with the Sips isotherm model with calculated maximum adsorption capacities very close to experimental values (1354.6 and 1365.5 mg/g, respectively). Significant dye uptakes suggest dicarboxymethyl cellulose can be used as an alternative to commercial adsorbents. Kinetics studies revealed experiments were well described by the pseudo second-order kinetic model which is associated with chemisorption processes. Filtration experiments using ultrafiltration membranes proved successful in removing methylene blue. Filtration with Microdyn Nadin UV150 and UC500 removed close to 90% of this cationic dye. Even though the membranes pore size was much larger than that of methylene blue, it was not permeated. Dye adsorption coupled with filtration was slightly more efficient in the removal of methylene blue (≈95%), but the process was much slower. Reusability studies showed that DCMC can be reused, whereas the membranes did not desorb the cationic dye. The reusability of the polymer can clearly contribute to a more sustainable and cost-effective process. Future work should address the use of DCMC in the treatment of real wastewater including scale-up experiments allowing a detailed cost-benefit analysis to assess commercial feasibility of the treatment proposed.

## Figures and Tables

**Table 1:** Conditions for dicarboxymethyl cellulose (DCMC) polymers production.

Sample	Molar Ratio AGU:BMA:NaOH	*m*_Cellulose_ (g)	*m*_BMA_ (g)	*m*_NaOH_ (g)
DCMC 1	1:1:3	5.0	6.9	3.7
DCMC 2	1:2:3	5.0	13.9	3.7
DCMC 3	1:3:3	5.0	20.9	3.7

**Table 2:** Determination of carboxylate groups on polymer samples produced.

Sample	*C*_Na_ (mg/L)	*m*_Na_ (mg) in Sample	*m*_pol_ (mg) in Sample	% Na	CG (mmol_Na_/g_pol_) ^1^
DCMC 1	9.69	0.0048	1.3	0.37	0.16
DCMC 2	10.45	0.0052	1.2	0.44	0.19
DCMC 3	12.43	0.0062	1.0	0.62	0.27

^1^ CG stands for carboxylate groups per gram of polymer.

**Table 3:** Dimensions of dicarboxymethyl cellulose.

Sample	Length (µm)	Surface Area (µm^2^)
DCMC 1	14.0 ± 4.5	207.7 ± 141.2
DCMC 2	77.5 ± 26.8	6284.1 ± 3948.6
DCMC 3	78.2 ± 21.5	7415.0 ± 2490.5

**Table 4:** Adsorption isotherms parameters.

Sample	Langmuir			Freundlich		
	*q_m_* (mg/g)	*K_D_* (L/mg)	*R* ^2^	*n*	*K* (L/mg)	*R* ^2^
DCMC 1	277.6 ± 12.0	0.021 ± 0.004	0.913	3.4 ± 0.9	0.037 ± 0.018	0.841
DCMC 2	546.2 ± 44.2	0.012 ± 0.004	0.938	3.0 ± 0.8	0.052 ± 0.029	0.789
DCMC 3	887.6 ± 107.4	0.014 ± 0.005	0.921	2.6 ± 0.7	0.071 ± 0.040	0.767

**Table 5:** Comparison of maximum adsorption capacity of methylene blue with other adsorbents.

Adsorbent	*q_m_* (mg/g)	pH	Reference
DCMC	1354.6	6.4	Present study
MCA–E0.7/CMC ^1^	998.2	7.0	[36]
CAC ^2^	980.3	7.4	[42]
*A. platensis* biomass ^3^	312.5	7.5	[9]
SCSM ^4^	178.6	7.0	[43]
KT3B ^5^	99.9	9.0	[44]
DCMC	887.6	3.0	Present study
CHACZ ^6^	463.0	4.0	[38]

^1^ Epichlorohydrin-crosslinked carboxymethyl cellulose microspheres treated with 0.7 mL of butanol and modified with monochloroacetic acid; ^2^ Commercial activated carbon; ^3^ *Arthrospira platensis* biomass; ^4^ Extraction residues of Salvia mitiorrziza Bge modificated with 1 M NaCO_3_; ^5^ Natural raw (Algerian) kaolin; ^6^ Corn husk activated by ZnCl_2_.

**Table 6:** Adsorption kinetics parameters.

*C* (mg/L)	pH	Pseudo First-Order			Pseudo Second-Order		
		*K*_1_ (min^−1^)	*q_m_* (mg/g)	*R* ^2^	*K*_2_ (mg g^−1^ min^−1^)	*q_m_* (mg/g)	*R* ^2^
4	3.0	0.682 ± 0.347	0.830 ± 0.062	0.857	0.931 ± 0.619	0.881 ± 0.064	0.898
	6.4	4.587 ± 0.534	3.116 ± 0.030	0.995	6.014 ± 2.204	3.134 ± 0.037	0.993

**Table 7:** Results from the filtration processes.

Sample	Dye Removal Efficiency (%)
UV150	89.8
UC500	93.4

**Table 8:** Results from the adsorption coupled with filtration processes.

Sample	Dye Removal Efficiency (%)	
	Adsorption	Filtration
UV150	63.5	94.9
UC500	61.2	95.6
